# Effect of Doping on the Electronic Structure of the Earth’s Lower Mantle Compounds: FeXO_3_ with X = C, Al, Si

**DOI:** 10.3390/ma15031080

**Published:** 2022-01-29

**Authors:** Evgeniy D. Chernov, Alexey A. Dyachenko, Alexey V. Lukoyanov

**Affiliations:** 1 M.N. Miheev Institute of Metal Physics of Ural Branch of Russian Academy of Sciences, 620108 Ekaterinburg, Russia; chernov_ed@imp.uran.ru (E.D.C.); dyachenko@imp.uran.ru (A.A.D.); 2 Institute of Physics and Technology, Ural Federal University, Mira, 19, 620002 Ekaterinburg, Russia; 3 Skolkovo Institute of Science and Technology, 121205 Moscow, Russia

**Keywords:** electronic structure, magnetic moments, phase transitions, first principles calculations

## Abstract

The effect of the mutual doping of C, Si, and Al atoms on the electronic structure and magnetic properties of FeXO_3_ (X = C, Al, Si) compounds, which are constituent compounds of the Earth’s lower mantle, was studied. In our first principles calculations, it was found that doping with carbon for both FeSiO_3_ and FeAlO_3_ leads to the transition of the compound from a half-metallic state to a metallic one. The values of the magnetic moments of Fe were obtained for pure and doped compounds. For the doped compounds, there is a tendency of the Fe magnetic moment to increase with the growth in the number of substituted ions in the case of replacing Si with C and Si for Al; on the contrary, in the case of replacing Al with C and Si, a decrease in the magnetic moment was revealed. For FeXO_3_ (X = C, Al, Si), the obtained magnetic moment values were found to be in a good agreement with the known experimental data.

## 1. Introduction

The Earth’s mantle contains a huge amount of minerals of various compositions, and most of it, usually called the Earth’s lower mantle, is mainly composed of silicate perovskite (Mg,Fe)(Si,Al)O_3_ [1]. According to modern concepts, the Earth’s lower mantle (from 650 to 2800 km distance from the Earth’s surface to the center along the radius, which corresponds to pressures of 23–135 GPa) consists mainly of iron-containing magnesium perovskite (bridgmanite) (Mg,Fe)SiO_3_ [2] (more than 70%) and ferropericlase (Mg,Fe)O (about 20%). The remaining 10% includes cubic perovskite CaSiO_3_ (about 5%), as well as a solid solution (Mg,Fe)SiO_3_·Al_2_O_3_, iron-containing carbonates (Mg,Fe)CO_3_, Ca-ferrite (NaAlSiO_4_) and other oxide phases containing Si, Ca, Na, K, Al and Fe [3]. These compounds were formed as a result of thermobaric reactions, and are under conditions of gigantic compression, which determines the features of their magnetic and electronic states. Over the past decades, a large number of experimental materials [1,4] and the results of theoretical calculations [5,6,7,8] have been accumulated. The result of the studies is the description of the properties of these iron-containing components of the mantle under conditions close to those of the Earth’s lower mantle [9,10,11,12,13,14].

A number of works have been conducted in recent years, in which the effect of deformations under high pressure compression on ferropericlase, bridgmanite and siderite has been studied experimentally [15,16] and theoretically [17]. In bridgmanite (Mg,Fe)(Si,Al)O_3_, ferropericlase and magnesium wustite (Mg,Fe)O, the chemical composition is strongly variable. In (Mg,Fe)O, magnesium replaces iron in the metal sublattice [18]; in (Mg,Fe)(Si,Al)O_3_, in addition to the replacement of Mg by Fe in the A sites of the perovskite structure, the presence of Al at the Si positions in the B sites is possible [19]. For FeAlO_3_, only a few research results can be found in the literature [20,21,22].

Iron-containing carbonates (Mg,Fe)CO_3_ can play an important role in the carbon cycle of the Earth’s mantle and the entire planet [23]. Therefore, their properties, including those under pressure, need an adequate description. Magnesite MgCO_3_ is isostructural with siderite FeCO_3_, and crystallizes in a trigonal structure, the samples of which are found in a wide range of compositions and concentrations, both in a natural state and synthesized. X-ray diffraction measurements under pressure showed that siderite in this structure is stable at high pressures up to 66 GPa, with a transition at pressures of about 45 GPa, which was studied both experimentally [24] and theoretically [25,26]. In this study, we consider in detail the effect of the mutual doping of X = C, Si and Al atoms on the electronic structure and the magnetic properties of the FeXO_3_ compounds.

## 2. Materials and Methods

**Figure 1:**
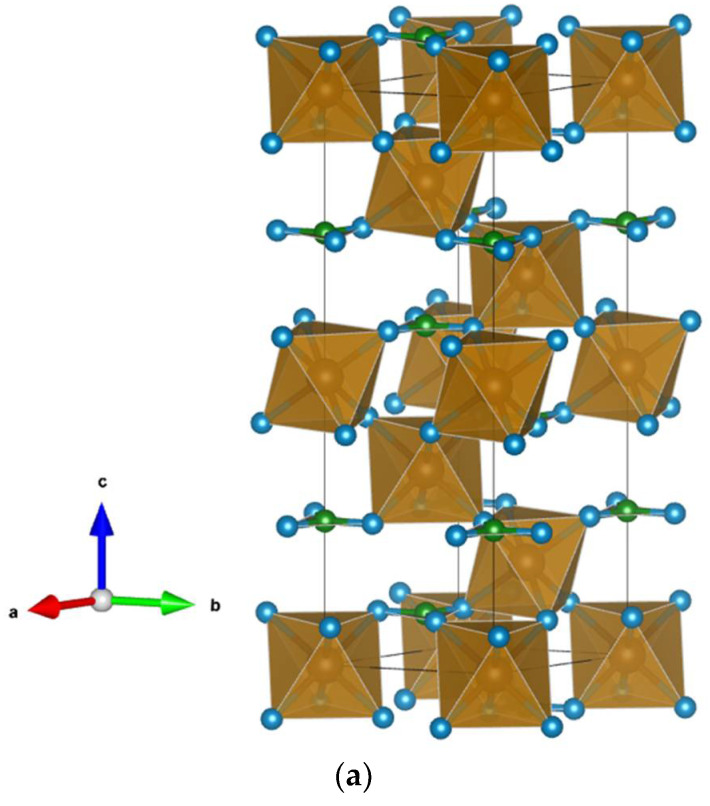

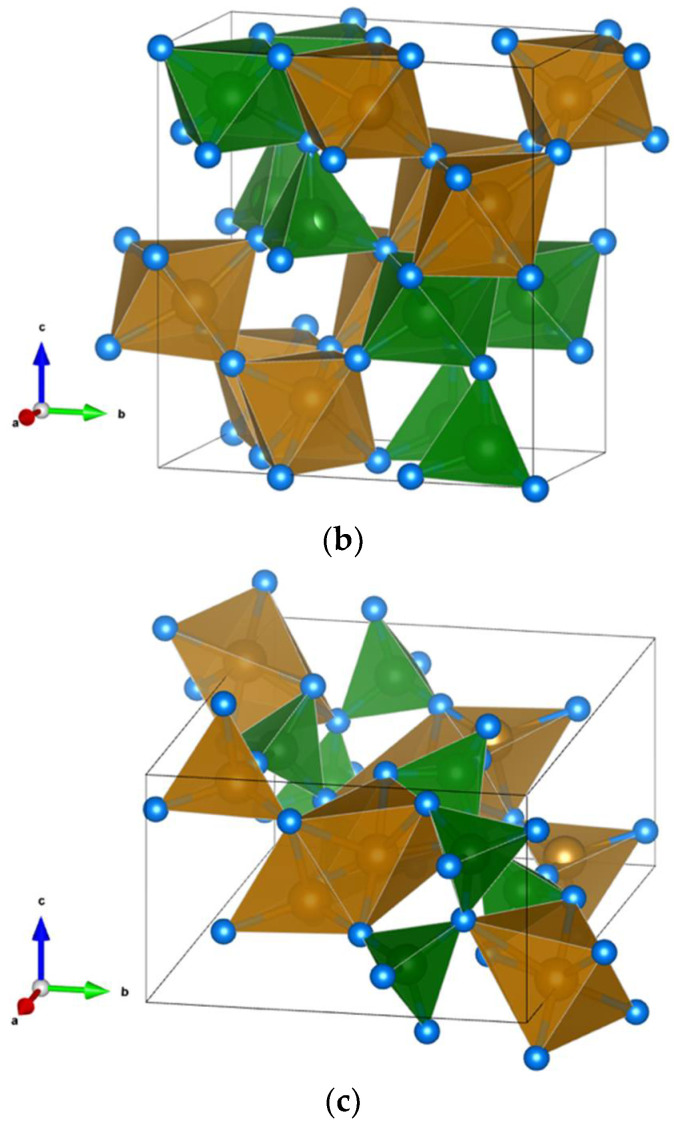
Crystal structure of: (**a**) FeCO_3_; (**b**) FeAlO_3_; (**c**) FeSiO_3_. Fe atoms are shown in brown, (C (**a**), Al (**b**), Si (**c**))—in green, O—in blue.

The FeCO_3_ compound has a trigonal R-3c symmetry group (number 167 in the list of crystallographic groups). The unit cell parameters are: a = b = 4.703 Å, c = 15.409 Å, *α* = *β* = 90°, *γ* = 120°. Fe ions occupy 4*c* positions (0, 0, 0), C ions are also in 4*c* positions (0, 0, 0.25) and O ions are in 4*c* positions with the coordinates (0.2818, 0, 0.25) [27]. The crystal structure of FeCO_3_ is plotted in Vesta [28] in Figure 1a. The unit cell of FeCO_3_ contains 2 iron atoms, 2 carbon atoms and 6 oxygen atoms. The Fe atom has an environment of six O atoms in the form of an octahedron, and the C atom has an environment of three O atoms in the form of a coplanar equilateral triangle. In this case, each O atom is adjacent to one C atom and two Fe atoms.

The FeAlO_3_ compound has an orthorhombic Pna2_1_ symmetry group (number 33 in the list of crystallographic groups). The unit cell parameters are: a = 4.984 Å, b = 8.554 Å, c = 9.241 Å, *α* = *β* = *γ* = 90° [21]. The illustration of the crystal structure of FeAlO_3_ is shown in Figure 1b. The cell contains 8 iron atoms (4 first-type Fe1 and 4 s Fe2), 8 aluminum atoms (4 first-type Al1 and 4 s-type Al2), and 24 oxygen atoms. Fe atoms of both types have an octahedral environment of six O atoms. The Al atom of the first type has an environment of four O atoms in the form of a tetrahedron, and the second type has an octahedron of O.

Additionally, and lastly, the FeSiO_3_ compound is investigated in the monoclinic phase P2_1_/c (number 14 in the list of crystallographic groups). The unit cell parameters are: a = 9.485 Å, b = 9.081 Å, c = 5.235 Å, *α* = *γ* = 90°, *β* = 103.207° [29]. The crystal structure of FeSiO_3_ is visualized in Figure 1c. The unit cell of FeSiO_3_ contains 8 iron atoms (4 first-type Fe1 and 4 second-type Fe2), 8 silicon atoms (4 first-type Si1 and 4 second-type Si2), and 24 oxygen atoms, following [7,8]. The Fe atom of the first type has an environment of six O atoms in the form of an octahedron, and that of the second type has a tetrahedron of O. Si atoms of both types have an environment of four O atoms in the form of a tetrahedron.

In this work, the electronic structure of the FeXO_3_ compounds was computed using the Quantum ESPRESSO software package [30,31]. This software package contains the most common basic and advanced exchange-correlation approximations and methods [30], as well as an impressive set of post-processing tools [31]. The exchange correlation potential was employed in a generalized gradient approximation (GGA) of Perdew–Burke–Ernzerhof (PBE) [32]. Wave functions were expanded in plane waves, Bloechl’s tetrahedron method was employed for Brillouin-zone integration on a 12 × 12 × 12 k-point mesh, and interactions between ions and valence electrons were taken into account within the framework of the method of augmented plane waves. A structural relaxation procedure was performed for the crystal structures to guarantee the lowest free energy of the systems. The calculations used the standard ultrasoft potentials from the pseudopotential library of Quantum ESPRESSO [33].

## 3. Results

**Figure 2:**
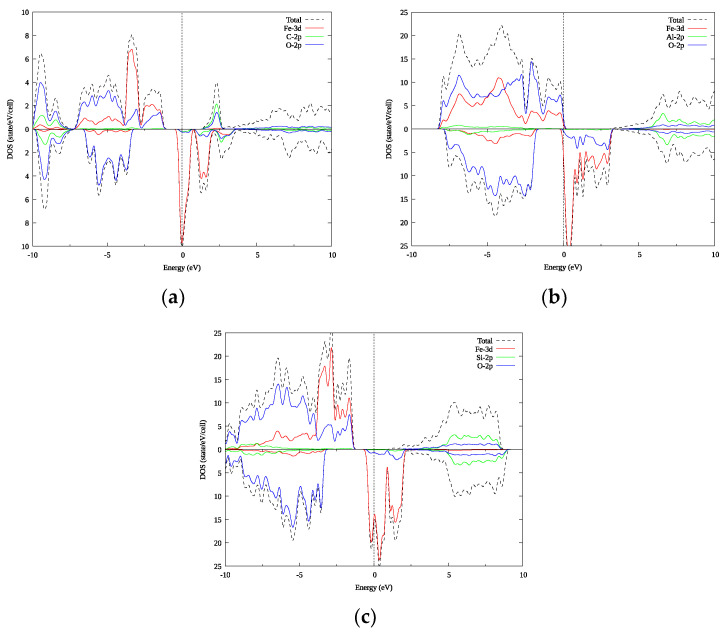
Densities of electronic states: (**a**) FeCO_3_; (**b**) FeAlO_3_; (**c**) FeSiO_3_. The plot is shifted relative to the Fermi energy, shown at zero as a vertical dashed line.

This section presents the results of first principles calculations of the electronic structures of the compounds FeCO_3_, FeAlO_3_ and FeSiO_3_. Density of state (DOS) plots for these compounds are shown in Figure 2a–c, respectively. The upper part of the plot corresponds to the majority spin projection and the lower part—to the minority spin projection. For the FeCO_3_ and FeSiO_3_ compounds, in the case of the majority spin projection, the Fermi level is located in the middle of the energy gap, and in the case of the minority spin projection, it is near the maximum of the density of states. In the conduction band, Figure 2, one can see the presence of a structure of several peaks at the Fermi energy, and above it, up to 3–3.5 eV, the peaks are mainly formed by the 3d electronic states of Fe; however, we also see that the 2p electronic states of O are involved in the formation of these peaks. 

**Figure A1:**
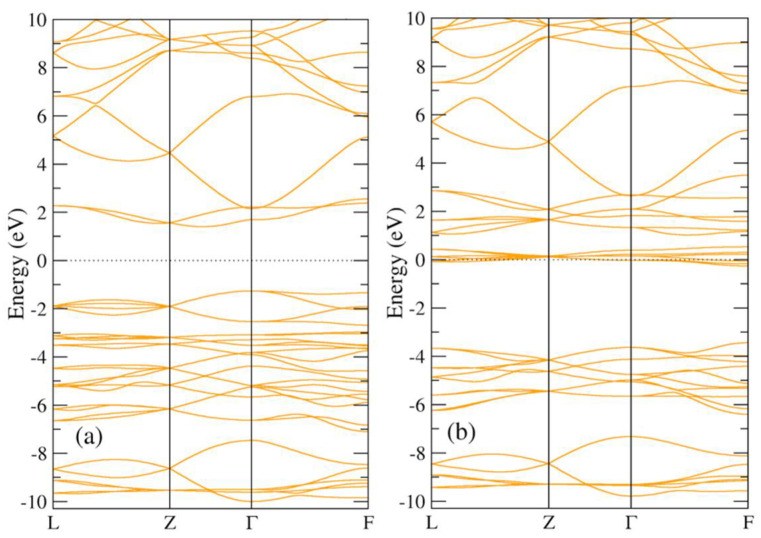
Band structure of FeCO_3_: (**a**) majority and (**b**) minority spin projections.

**Figure A2:**
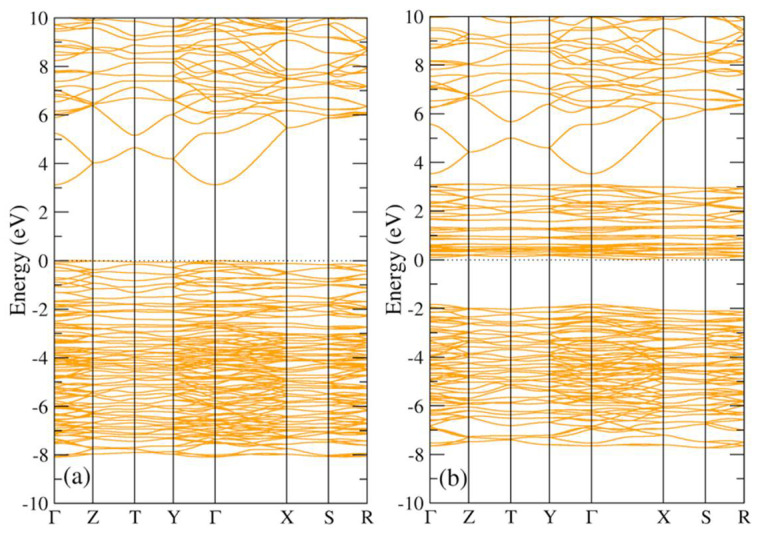
Band structure of FeAlO_3_: (**a**) majority and (**b**) minority spin projections.

**Figure A3:**
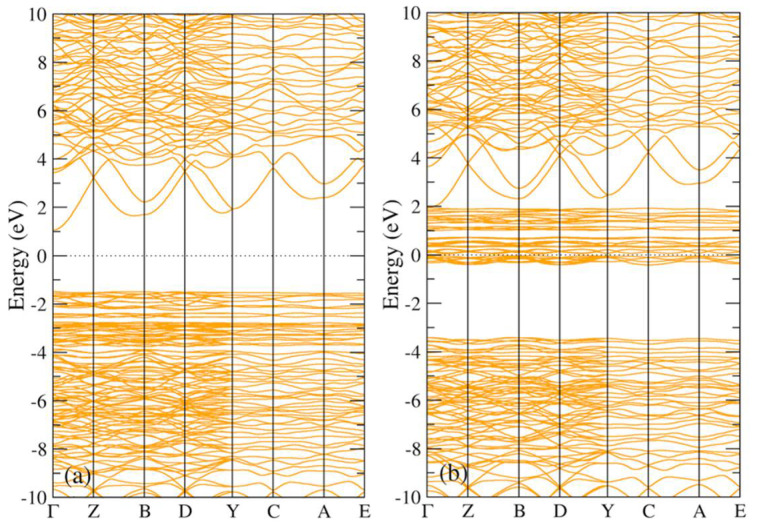
Band structure of FeSiO_3_: (**a**) majority and (**b**) minority spin projections.

In all three compounds, the valence band for both spin projections is formed by predominantly the oxygen states, with the occupied Fe 3d states in the majority spin projection, Figure 2. The p states of silicon and aluminum in the corresponding compounds lie mainly at a distance, starting from +5 eV relative to the Fermi level for both spin directions. For FeCO_3_ (see Figure 2a), the majority spin projection in the lower part of the conduction band exhibits a peak from a mixture of the 2p states of carbon and 2p states of oxygen, which is absent in the other two compounds. These results are in agreement with the known results from the literature [22,34,35]. In Appendix A, the corresponding band structures of FeCO_3_ are shown in Figure A1; for FeAlO_3_, in Figure A2; and for FeSiO_3_, in Figure A3, for (a) majority and (b) minority spin projections. The plots are shifted relative to the Fermi energy shown at zero as a horizontal dashed line. In the results of our work, in FeCO_3_, in the majority spin projection, the semi-metallic gap is 2.67 eV; in FeAlO_3_, in the minority spin projection, the half-metallic gap is 1.84 eV; and in FeSiO_3_, in the majority spin projection, the half-metallic gap is 2.54 eV. The large-scale benchmark of PBE on 472 compounds with a band gap [36] resulted in an error below 1 eV for the band gap, which is well below the calculated values. Obtaining an accurate band gap calculation/prediction is a major problem for all DFT approximations and methods, and it is extensively investigated and reviewed, e.g., [36,37,38]. A number of methods and approaches are being proposed to tackle this problem, e.g., [39,40,41,42,43]. On the other hand, most sophisticated methods are either parameter dependent, or demand tremendous computational resources to handle large cells [44,45,46].

In addition to the densities of states, the values of the magnetic moments of atoms are also of interest. The magnitude of the magnetic moment of an atom can be obtained experimentally; therefore, it is possible to compare the results of the calculations with the known experimental values. Table 1 shows the values of the magnetic moments for the pure FeCO_3_, FeSiO_3_ and FeAlO_3_ compounds. These are the extreme values for all combinations of the substitution of one atom (C, Al, Si) by another, which are discussed below. The magnetic moment of oxygen for any combination can be expected to be between 0.02 and 0.64 μ_B_. 

In addition to FeXO_3_ with X = (C, Al, Si), which is discussed above, the compounds with the substituted X atoms of one type for another, which are of the main interest of this study, were considered. The unit cells of FeSiO_3_ and FeAlO_3_ contain eight Si/Al atoms of two types (each type has a different environment). In this work, all possible options for doping up to half of the substituted atoms were considered, i.e., compounds obtained by the substitution in the initial crystal lattice of FeSiO_3_ and FeAlO_3_ with one to four atoms of Si and Al, respectively. These can be represented as the composition Fe_8_X^A^_4-X_ Y^A^_X_ X^B^_4-X_Y_y_^B^O_24_, where X^A(B)^ is the initial atom of type A (B), Y^A^ is the substituted atom of type A (B) and x (y) is the number of substituted atoms of type A (B). The X atom can be Si or Al, the Y atom is Si, Al, or C, while X is not equal to Y, which defines the large number of calculations based on the possible C, Al, Si substitutions.

### 3.1. Doped FeCO_3_

**Figure 3:**
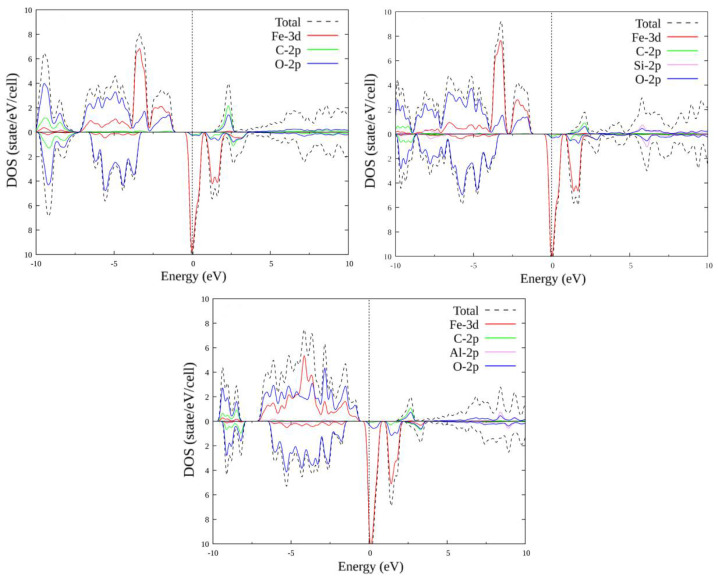
Total and partial densities of states for: FeCO_3_; FeC_0.5_Si_0.5_O_3_; FeC_0.5_Al_0.5_O_3_.

The unit cell of FeCO_3_ contains two C atoms of the same type (with a symmetrical arrangement); therefore, there is only one nonequivalent option for replacing C with another atom. Al and Si are substituted atoms. One of the C atoms was replaced by Al and Si, respectively. The self-consistent calculations were performed, and the plots of the total and partial densities of states were obtained. A partial substitution of aluminum for carbon (Figure 3) for the minority spin projection decreases the distance between the band below the Fermi level, formed by the O states, and the band at the Fermi level, consisting predominantly of the 3d iron states. For the majority spin projection, a greater mixing of the Fe and O states in the valence band is observed. In this case, the peak above the Fermi level, consisting of the 2p states of carbon and oxygen, decreases. In this case, the states of aluminum do not appear close to the Fermi level, causing a redistribution of density for the other atoms.

A partial substitution of silicon for carbon has minimal effect on the electronic states with the minority spin projection. With aluminum doping, the peak of a mixture of the carbon and oxygen electronic states near +6 eV decreases. The silicon states themselves begin to appear above +5 eV relative to the Fermi level.

### 3.2. Doped FeAlO_3_

**Figure 4:**
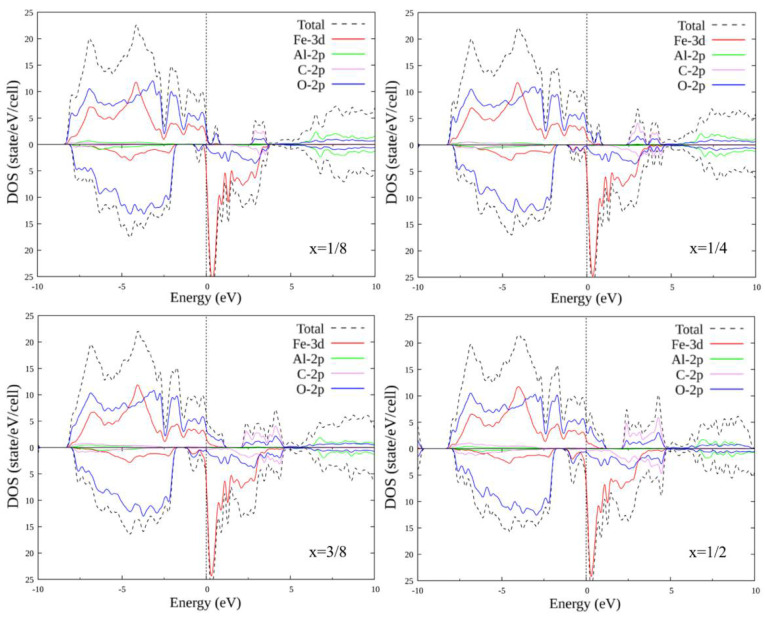
Densities of states FeAl_1-x_C_x_O_3_, where x = 1/8; 1/4; 3/8; 1/2. Replacement configurations (1, 0), (1, 1), (2, 1) and (2, 2) are selected.

**Figure 5:**
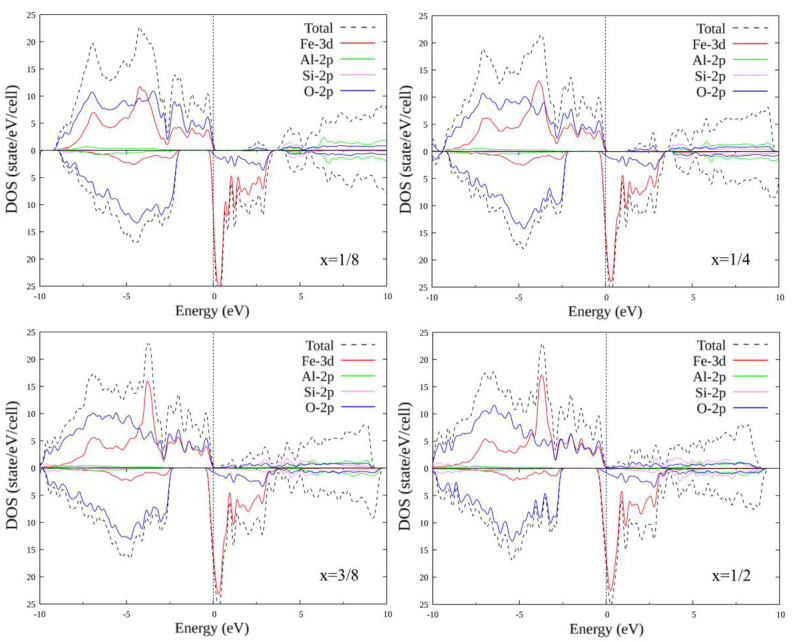
Densities of states FeAl_1-x_Si_x_O_3_, where x = 1/8; 1/4; 3/8; 1/2. Replacement configurations (1, 0), (1, 1), (2, 1) and (2, 2) are selected.

The unit cell of FeAlO_3_ contains four Al atoms of one type and four Al atoms of the second type (with a different environment). As in the case of FeSiO_3_, we consider substitutions of only one to four Al atoms (different combinations of different types of Al1 and Al2). In total, there are 14 different options for replacing one to four Al atoms with another atom. Self-consistent calculations were performed, and plots of the total and partial densities of states were plotted for all substitution options. Some of the plots for configurations (1, 0), (1, 1), (2, 1) and (2, 2) are shown in Figure 4 and Figure 5. The plots within each set with the same number of replaced atoms have insignificant differences; therefore, their consideration is omitted.

When Al atoms are replaced by C atoms, the energy gap for the majority spin projection disappears: the compound transitions from the semi-metallic state to the metallic one. An oxygen peak appears just above the Fermi level, which then merges with the valence band, creating a density of states at the Fermi level. Above the energy scale, a region of carbon–oxygen states is formed, which splits and increases in size with an increase in the number of substituted atoms. For the minority spin below the Fermi level, a small peak also appears, including the states of oxygen and iron, which also increases in size. 

When Al atoms are replaced by Si atoms for the majority spin projection, the energy gap decreases in size, but, in contrast to substitution by C, it does not disappear completely. A small oxygen and silicon region appears above the Fermi level, and increases in size depending on the concentration of the substituent. For minority spin, the distribution of states remains practically unchanged. 

### 3.3. Doped FeSiO_3_

The unit cell of FeSiO_3_ contains four Si atoms of one type and four Si atoms of the second type (with a different environment). It makes sense to consider the substitutions of only one to four Si atoms (different combinations of different types of Si1 and Si2), since the substitution of a larger number of atoms already corresponds to symmetrical substitution in compounds FeCO_3_ and FeAlO_3_ for C and Al by Si, respectively. In total, there are 14 different options for replacing one to four Si atoms with another atom: (0, 1), (0, 2), (0, 3), (0, 4), (1, 0), (1, 1), (1, 2), (1, 3), (2, 0), (2, 1), (2, 2), (3, 0), (3, 1) and (4, 0), where the record (x, y) is a pair of numbers: x is the number of Si1 atoms replaced by X atoms, and y is the number of Si2 atoms replaced by X atoms, where X = C, Al. 

**Figure 6:**
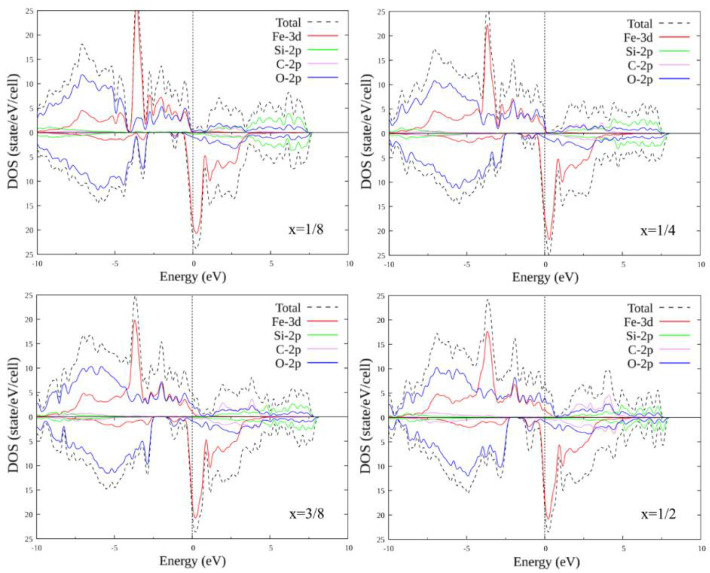
Densities of states FeSi_1-x_C_x_O_3_, where x = 1/8; 1/4; 3/8; 1/2. Replacement configurations (1, 0), (1, 1), (2, 1) and (2, 2) are selected.

**Figure 7:**
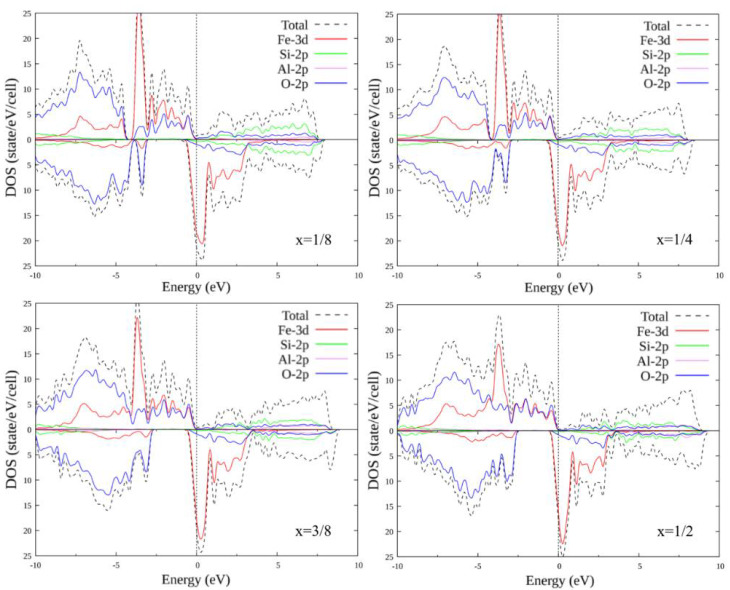
Densities of states FeSi_1-x_Al_x_O_3_, where x = 1/8; 1/4; 3/8; 1/2. Replacement configurations (1, 0), (1, 1), (2, 1), and (2, 2) are selected.

In similarity with the pure compound, self-consistent calculations were performed, and the plots of the total and partial densities of states were plotted for FeSiO_3_ with all substitution options. Below is only a part of the plots, for configurations (1, 0), (1, 1), (2, 1) and (2, 2) (Figure 6 and Figure 7). The plots within each set with the same number of replaced atoms have insignificant differences; therefore, their consideration is omitted. When Si atoms are replaced by C atoms, the energy gap for the majority spin projection first decreases and eventually disappears completely: the compound transitions from the half-metallic state to the metallic one. A carbon + oxygen + silicon cluster appears, in which the density of the state of carbon increases with an increase in the number of replaced atoms. For the minority spin states below the Fermi level, a small peak appears, including a mixture of the oxygen and iron states. With an increase in the number of the replaced atoms, it first splits into two parts, then merges again and increases in size. 

With the substitution of Al atoms for Si atoms, the energy gap for the majority spin projection, as in the case of substitution with C, first decreases, but then increases to several tenths of an eV. Otherwise, no significant changes were observed in the growth of the number of the substituted atoms.

Thus, we analyzed the electronic structure of compounds FeCO_3_, FeAlO_3_ and FeSiO_3_ without doping, as well as with doping, examining the substitution of C for Si and Al in FeCO_3_, the substitution of Si for C and Al in FeSiO_3_, the substitution of Al for C and Si in FeAlO_3_. It was found that doping with carbon for both FeSiO_3_ and FeAlO_3_ leads to the appearance of a nonzero density of states at the Fermi level for the majority spin projection, which should correspond to the transition of the compound from the semi-metallic state to the metallic one. For the other cases, the effect of doping on the electronic structure is less pronounced. 

## 4. Discussion

In addition to the densities of states, the values of the magnetic moments of atoms are also of interest, since information about this is the basis for understanding the magnetic properties of the compounds under study. The magnitude of the magnetic moment of an atom can be obtained experimentally; therefore, it is possible to compare the results of calculations carried out in this work with the known experimental values for the compounds FeCO_3_, FeAlO_3_ and FeSiO_3_. 

When carbon is replaced by silicon (in Table 2, Fe_2_CSiO_6_ is compared to Fe_2_C_2_O_6_), the magnetic moments change only slightly, in contrast to the changes caused by replacing carbon with aluminum (in Table 2, Fe_2_CAlO_6_ is compared to Fe_2_C_2_O_6_). This follows from the similarity of the chemical properties of carbon and silicon, as they are members of the same group in the periodic table. From Table 1 and Table 2, it follows that the magnetic moment of oxygen is an order of magnitude less than that of iron, and the other ions by two orders of magnitude. Therefore, below, in Table 3 and Table 4, only the magnetic moment of iron is considered.

For the Fe_8_Si_8_O_24_ and Fe_8_Al_8_O_24_ compounds, the substitution combinations are significantly larger than for Fe_2_C_2_O_6_, since they include more atoms in the unit cell. In addition, the compounds contain two types of Si and Al atoms, respectively, depending on their position in the cell. For example, for Fe_8_Si_8_O_24_, the number of nonequivalent substitutions of Si for C will be 14, with the substitution of one Si1 atom, one Si2 atom, two Si1 atoms, one Si1 atom and one Si2 atom, etc. Therefore, in Table 3 and Table 4, the averaged values and their spread are presented. 

With an increase in the number of substituted atoms both in the case of replacing silicon with carbon, and in the case of replacing silicon with aluminum, an increase in the value of the magnetic moment is observed. On the contrary, in the case of replacing aluminum with carbon and silicon, a decrease in the magnitude of the magnetic moment is observed. This trend persists, taking into account the deviation of the magnetic moments for various types of the iron ions.

The results for silicon and aluminum coincide, for a configuration with the replacement of half of the silicon atoms by aluminum atoms, and for a configuration symmetrical to it, with the replacement of half of the aluminum atoms by silicon atoms. This confirms that the calculations were carried out correctly. A comparison of the obtained values of the magnetic moment of iron for the compounds with the known experimental data and previous calculations is presented in Table 5. In the case of FeCO_3_ and FeAlO_3_, the experimental values were obtained by neutron diffraction, and in the case of FeSiO_3_, the method of Mössbauer spectroscopy was used, together with a SQUID magnetometer. For FeCO_3_, the calculated magnetic moment of iron obtained in this work is closer to an experimental value than the previous calculation [47]. For FeAlO_3_, the magnetic moment of iron reported in [20] was calculated for another crystal structure, namely, a perovskite structure. It should also be noted that the experimental value in [21] was obtained for polycrystalline samples of FeAlO_3_. For FeSiO_3_, the DFT + DMFT method was employed in [7], and then the difference between the calculated value obtained in this work and the results of the other calculations [7,20] can be attributed to the difference in methods and approximations employed.

## 5. Conclusions

In this work, we investigated the electronic structure of the iron oxide compounds FeCO_3_, FeAlO_3_ and FeSiO_3_, which are found in the Earth’s lower mantle in a pure, doped or admixed state. The first principles calculations were carried out for the pure and doped compounds. For compounds with doping, a comparative analysis of the electronic states and their distribution were performed. It was found that doping with carbon for both FeSiO_3_ and FeAlO_3_ leads to the transition of the compound from the half-metallic state to the metallic one. For the other cases, the effect of doping on the electronic structure is less pronounced. In our theoretical calculations, the values of the magnetic moments of Fe were obtained for pure and doped compounds. For pure compounds, agreement with the experimental values is observed with an accuracy of 10%. For the doped compounds, there is a tendency of the Fe magnetic moment to increase with the growth in the number of substituted ions in the case of replacing Si with C and Si for Al; on the contrary, in the case of replacing Al with C and Si, a decrease in the magnetic moment was revealed. This study contributes to the general body of knowledge about the properties of compounds that are widely present in the Earth’s lower mantle. 

## Figures and Tables

**Table 1:** Magnetic moments of individual ions in FeXO_3_ compounds for X = C, Al, Si.

Ions	FeCO_3_, μ_B_	FeAlO_3_, μ_B_	FeSiO_3_, μ_B_
Fe1	3.64	3.64	3.97
Fe2	3.64	3.58	3.98
X	0.015	0.014–0.016	0.014–0.016
O	0.11	0.02–0.18	0.12–0.64

**Table 2:** Magnetic moments (in μ_B_) of ions in Fe_2_CXO_6_ compounds (X = C, Al, Si): pure Fe_2_C_2_O_6_ and doped compounds with substitution of C for Si and Al, respectively.

Ions	Fe_2_C_2_O_6_	Fe_2_CSiO_6_	Fe_2_CAlO_6_
Fe	3.64	3.65	3.89
C	0.015	0.010	0.030
X	0.015	0.009	0.0026
O	0.11	0.10–0.11	0.13–0.26

**Table 3:** Magnetic moments (in μ_B_) of the Fe ion in pure Fe_8_Al_8_O_24_ and doped compounds with substitution of Al for C and Si, respectively.

Fe_8_Al_8−n_X_n_O_24_	n = 0	n = 1	n = 2	n = 3	n = 4
X = C	3.98 ± 0.01	3.92 ± 0.06	3.87 ± 0.10	3.82 ± 0.13	3.77 ± 0.13
X = Si	3.98 ± 0.01	3.95 ± 0.04	3.90 ± 0.07	3.84 ± 0.09	3.78 ± 0.09

**Table 4:** Magnetic moments (in μ_B_) of the Fe ion in pure Fe_8_Si_8_O_24_ and doped compounds with substitution of Si for C and Al, respectively.

Fe_8_Si_8−n_X_n_O_24_	n = 0	n = 1	n = 2	n = 3	n = 4
X = C	3.63 ± 0.04	3.65 ± 0.10	3.69 ± 0.09	3.72 ± 0.07	3.71 ± 0.07
X = Si	3.63 ± 0.04	3.62 ± 0.10	3.67 ± 0.10	3.72 ± 0.11	3.78 ± 0.09

**Table 5:** Comparison of the magnetic moments (in μ_B_) of the Fe ion in the FeCO_3_, FeAlO_3_ and FeSiO_3_ obtained theoretically in our work, as a result of previous calculations, and experimental values.

Compound	This Work	Previous Calculations	Experiment
FeCO_3_	3.64	3.71 [47]	3.61 [47]
FeAlO_3_	3.97	3.69 [20]	3.4 ± 0.3 [21]
FeSiO_3_	3.67	3.8 ± 0.1 [7]	4.0 ± 0.1 [48]

## Data Availability

The data presented in this study are available on request from the corresponding author.
